# Methicillin-resistant *Staphylococcus aureus* Skin Infections

**DOI:** 10.3201/eid1110.050680

**Published:** 2005-10

**Authors:** Jui-Shan Ma

**Affiliations:** * Show-Chwan Memorial Hospital, Changhua, Taiwan

**Keywords:** MRSA, skin infections, drainage, resistance, letter

**To the Editor:** Moran et al. write, "In areas with a high prevalence of CA-MRSA [community acquired methicillin-resistant *Staphylococcus aureus*], empiric treatment for skin and soft tissue infections (SSTIs) with β-lactam agents such as cephalexin may no longer be appropriate. Oral agents such as clindamycin or trimethoprim/sulfamethoxazole and rifampin should be considered in CA-MRSA" (*1*). However, some studies have had different results. Lee et al. reported that 31 (84%) of 37 Texas children with CA-MRSA SSTIs showed clinical improvement after incision and drainage, even though they received an "ineffective" antimicrobial agent that was not changed after the susceptibility results became available (*2*). These researchers also reviewed some reports with similar experience in the United States and further suggested that incision and drainage without adjunctive antimicrobial therapy were effective in immunocompetent children for CA-MRSA SSTIs <5 cm in diameter.

Several studies on Taiwanese children with CA-MRSA SSTIs agree with the viewpoint of Lee et al. Chen and colleagues reported that 22 (63%) of 35 episodes of CA-MRSA superficial soft tissue infections in children were cured by nonsusceptible antimicrobial therapy, regardless of surgical intervention (*3*). In a study by Wang et al., oxacillin, with or without incision and drainage, was effective in 16 (89%) of 18 children with CA-MRSA SSTIs, even in a case with high-level oxacillin resistance (MIC>8 μg/mL) (*4*). Fang et al. also reported that 16 (55%) of 29 children with CA-MRSA SSTIs were eventually cured with therapy to which their infections were not susceptible (*5*). With these experiences and concerns about the growing problem of bacterial resistance, we suggest that incision and drainage, with or without adjunctive antimicrobial therapy, are adequate to treat noninvasive CA-MRSA SSTIs in immunocompetent children and that oxacillin or first-generation cephalosporins are still effective and sufficient under such conditions. Vancomycin and other agents that are effective against MRSA isolates should be reserved for invasive CA-MRSA infections or for immunocompromised patients. Although Moran's study was focused on adults, not on children as these studies were, we believe these suggestions are also appropriate when applied to CA-MRSA SSTIs in adults.

Finally, the antibiogram of CA-MRSA isolates may vary from country to country. In Taiwan, CA-MRSA isolates are also resistant to multiple antimicrobial agents; 71.4%, 91.4%, and 41.2% are resistant to clindamycin, erythromycin, and chloramphenicol, respectively (*4*). Trimethoprim/sulfamethoxazole is more effective against CA-MRSA isolates than other first-line antimicrobial agents: the resistance rate is 0%–65.7% (*4**,**5*). Therefore, clindamycin and trimethoprim/sulfamethoxazole may be not adequate empiric antimicrobial agents for SSTIs in Taiwan or other areas with a high prevalence of CA-MRSA.
