# Supplementary material for: Methicillin-resistant Staphylococcus aureus Skin Infections
Source: Emerg Infect Dis. 2005 Oct;11(10):1644–5. doi: 10.3201/eid1110.050680 (PMC3366755; doi:10.3201/eid1110.050680)
Supplement: Related texts

# Reply

**DOI:** 10.3201/eid1110.050858

**Authors:** Gregory J. Moran, Ricky N. Amii, Frederick M. Abrahamian, David A. Talan

**Affiliations:** * Olive View–UCLA Medical Center, Sylmar, California, USA

**In response:** Dr Ma makes an excellent point about the limitations of study data on antimicrobial drug treatment of skin abscesses (*1*). All of the patients described in our study (*2*) required antimicrobial drug therapy, and most were admitted to the hospital. However, we did not mean to imply that all skin abscesses require antimicrobial drug treatment. Our own practice is to give antimicrobial drug therapy only when a skin abscess is associated with definite surrounding cellulitis, systemic signs, or both. Although various criteria have been published, in practice this is a judgment call, and we suspect that physicians vary considerably in use of antimicrobial agents for skin infections.

Because most cellulitis associated with skin abscess will improve with adequate drainage, designing a study that will find a difference in outcome attributable to the antimicrobial drug is difficult. More studies are needed to determine whether antimicrobial agents with in vitro activity against methicillin-resistant *Staphylococcus aureus* (MRSA) are more clinically effective than those lacking such activity. Perhaps these studies should focus on those infections for which antimicrobial agents would be expected to have the greatest impact (e.g., infected wounds with cellulitis), rather than abscesses that can be expected to improve with incision and drainage alone.

When the decision is made to use an antimicrobial agent, it is difficult to justify choosing one to which the infecting organism will likely be resistant. Because MRSA is now the most common cause of skin infections at our institution, we choose agents with activity against the MRSA strains in our community. We do not believe that choosing an antimicrobial agent to which the infecting organism is susceptible is more likely to contribute to the general problem of antimicrobial drug resistance.

## References

[1] Ma J-S. Methicillin-resistant *Staphylococcus aureus* skin infections [letter]. Emerg Infect Dis. 2005;11:1644–5. 10.3201/eid1110.05068016355509PMC3366755

[2] Moran GJ, Amii RN, Abrahamian FM, Talan DA. Methicillin-resistant *Staphylococcus aureus* in community-acquired skin infections. Emerg Infect Dis. 2005;11:928–30. 10.3201/eid1110.05085815963289PMC3367577
